# The reaggregation of normal granulosa-cumulus cells and mouse oocytes with polycystic ovarian syndrome in vitro: An experimental study

**DOI:** 10.18502/ijrm.v19i11.9914

**Published:** 2021-12-13

**Authors:** Amaneh Moradi, Fatemeh Ghasemian, Farhad Mashayekhi

**Affiliations:** Department of Biology, Faculty of Sciences, University of Guilan, Rasht, Iran.

**Keywords:** PCOS, Co-culture, Granulosa-cumulus cells, IVM, Cx43.

## Abstract

**Background:**

The dialogue between oocytes and their surrounding cells plays a major role in the progress of oocyte meiosis and their developmental potential.

**Objective:**

This study aimed to evaluate the effect of co-culture of normal granulosa-cumulus cells (GCCs) with oocytes from polycystic ovarian syndrome (PCOS) mice.

**Materials and Methods:**

Normal GCCs were collected from 10 virgin adult Naval Medical Research Institute female mice (30-35 gr, 7-8 wk old), and were cultured in an alpha-minimum essential medium supplemented with 5% fetal calf serum for 24-48 hr (1 × 10 ${}^{6}$ cells/well). Then, germinal-vesicle oocytes from PCOS mice were cultured in the presence of cultured normal GCCs (experimental group) and without GCCs (control group). The maturation rate and quality of the PCOS oocytes were examined by evaluating *TFAM* and *Cx43* gene expression (real-time PCR) and the connection among PCOS oocytes and normal GCCs after 24 hr of culture.

**Results:**

The co-culture of normal GCCs and PCOS oocytes in the experimental group led to the formation of a complex called a PCOS oocyte-normal GCCs complex. The maturation rate of these complexes was significantly increased compared to that of the control group (p ≤ 0.001). A significant difference was also found in the expression of *Cx43* (p ≤ 0.001) and *TFAM* (p < 0.05) genes in the experimental group compared with the control group. The connection between PCOS oocytes and normal GCCs was observed in the scanning electron microscope images.

**Conclusion:**

Co-culture with normal GCCs improves the capacity of PCOS oocytes to enter meiosis, which may result in the promotion of assisted reproduction techniques.

## 1. Introduction 

Maturation and developmental competence of oocytes and their quality are important criteria in successful reproductive outcomes of assisted reproduction technologies. Polycystic ovarian syndrome (PCOS) is known as the most common cause of female infertility during reproductive age. It is distinguished by oligomenorrhea, bilateral polycystic ovaries, hyperinsulinemia, biochemical and/or hyperandrogenism, and oligo- or chronic anovulatory (1-3). One of the most important symptoms in PCOS patients is follicular development arrest and dysregulation of paracrine follicle activity (4). As a result, one of the main important concerns in PCOS patients is the poor competence of oocyte development (3, 5) and weak response to ovarian stimulation (6, 7). For this reason, the use of assisted reproductive technologies such as in vitro maturation (IVM) for such patients is suggested.

During the IVM procedure, the maturation medium usually replaces the ovarian follicular environment. Therefore, it is necessary to create an appropriate culture medium for improving the competence of immature oocyte development for fertilization and embryogenesis (7). The development and growth of oocytes are supported by granulosa cells (GCs) from primordial to antral stages (8); therefore, one of the strategies to increase in vitro oocyte maturation is co-culture with GCs (9).

In a study, the developmental rate of porcine oocytes derived from early antral follicles improved using reconstructed oocyte-GC complexes (10). In another study, the maturation and development competence of buffalo oocytes derived from preantral follicles was evaluated in the presence of antral follicles and the survival and growth of the preantral follicles improved in this co-culture system (11).

Many studies have reported oocytes as an avascular environment. Normal folliculogenesis relies on the crosstalk among GCs, cumulus cells, and oocytes via gap junction intercellular communication (12, 13). Two matchable hemichannels (connexin, Cx) in the plasma membrane of adjacent cells are involved in the formation of gap junctions. *Cx43* is mainly localized to the membranes of the GCs and cumulus cells (12). It has been reported that *Cx43* expression decreases in the presence of high androgen levels and hence gap-junctional intercellular communication between human GCs is impaired (14). GCs in PCOS patients were induced to produce more androgens, which resulted in the enhancement of hyperandrogenemia and anovulation (1). High androgen levels may also impair folliculogenesis in PCOS patients (14). Therefore, the co-culture of PCOS oocytes with normal cumulus GCs may improve the maturation rate and competence of oocyte development.

To the best of our knowledge, there have been no published studies on the use of normal cumulus GCs to improve the maturation of PCOS oocytes in co-culture conditions. Therefore, the purpose of this study was to answer the following questions: (i) Will a connection be made between normal cumulus-GCs and PCOS immature oocytes in a co-culture medium? (ii) How will a connection between PCOS immature oocytes and normal granulosa-cumulus cells (GCCs) affect the maturation rate of PCOS oocytes and their quality?

## 2. Materials and Methods

### Study animals 

This experimental study was conducted over a period of two years (2019-2020) in the University of Guilan, Rasht, Iran. 20 virgin adult Naval Medical Research Institute female mice (30-35 gr, 7-8 wk old) were used for the study. Animals were acquired from the Razi Institute (Karaj, Iran) and housed in a central animal care room with a controlled environment of 22 ± 3°C temperature, 45-55% humidity and a 12 hr light/dark cycle. Mice were kept in a cage in groups of four and fed with a standard diet and water accessed ad libitum. All chemicals and reagents were purchased from Sigma Aldrich Company (Germany), unless otherwise specified.

### PCOS induction 

All the experimental animals except for those in the control group were injected with estradiol valerate (Aburaihan Co., Iran) at a dose of 40 mg/kg body weight dissolved in 0.5% sesame oil by intramuscular injection once daily for 60 days. Vaginal epithelia smears were obtained daily and evaluated by light microscope (Olympus, Japan) using Giemsa stain to determine the induction of PCOS. An irregular estrous cycle and occurrence of persistent vaginal cornification phase were the symptoms of PCOS induction. Ovaries were cut through at their longest longitudinal dimension and fixed in alcoholic Bouin's solution. After dehydration, the ovaries were serially sectioned at 5 µm, and stained with hematoxylin and eosin. The sections were used for histologic evaluation of the PCOS ovaries.

In addition, to confirm the PCOS induction, blood samples of PCOS mice were collected transcardially. Then, the separated serum was stored at -20°C. Using immunofluorometric techniques, the levels of serum follicle-stimulating and luteinizing hormones were measured. 2.9% and 2.6% were considered as total assay variation coefficients. In addition, the Coat-A-Count RIA kit was used to analyze the level of serum testosterone with a sensitivity of 4 ng/dL (0.139 nmol/L).

### Experimental design

To produce a conditioned medium, normal GCCs were collected from the ovaries of normal mice. Following cervical dislocation, the ovaries were placed in α-minimum essential medium (Gibco, Paisley, UK) supplemented with 5% fetal bovine serum (FBS; Gibco, UK), and were mechanically dissected. GCCs were collected and cultured in 24-well plates (1 × 10 ${}^{6}$ cells/well) containing 500 μl of the same medium supplemented with 5% FBS, 50 μg/ml penicillin, and 50 μg/ml streptomycin for 24-48 hr. The unattached cells were removed following replacement with fresh medium.

**Figure 1:**
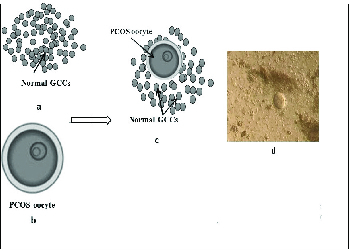
Process of PONC complexes formation, a) The normal GCCs were cultured for 24-48 hr, b) Then, the PCOS oocytes were added to them for another 24 hr, c) The PONC complexes were observed after culture, d) Co-culture of PCOS immature oocytes and normal GCs ( × 40 magnification).

After the confirmation of PCOS induction and following excision of the ovaries, the PCOS ovaries were placed in an α-minimum essential medium supplemented with 5% FBS. The ovaries were mechanically dissected and oocytes at the germinal vesicle stage were collected. The mouse germinal vesicle oocytes showed a large spherical germinal vesicle (30 µm in diameter) and a single nucleolus. After washing three times, the co-culture of PCOS oocytes with cultured GCCs (one oocyte/well) were initiated in the α-minimum essential medium supplemented with 5% FBS, 0.23 mM sodium pyruvate, 75 mU/ml of follicle-stimulating hormone, 7.5 IU/ml human chorionic gonadotropin, 50 μg/ml penicillin, and 50 μg/ml streptomycin (experimental group). After 24 hr co-culture, a complex between PCOS oocytes with normal GCCs was observed and was referred to as a PCOS oocyte-normal GCC complex (PONC) (Figure 1). Therefore, the groups of study were as follows: 1) experimental group - PCOS oocytes cultured in the presence of normal GCCs; and 2) control group - PCOS oocytes cultured in the absence of normal GCCs.

The development of PONC complexes and the properties of the oocytes in these complexes were evaluated 24 hr after co-culture and compared to the control group. The maturation rate of the reconstructed complexes (the presence of the first polar body extrusion) was also examined 24 hr after co-culture (15). The GCCs' viability was assessed by trypan blue staining. The experiment was repeated five times.

### Scanning electron microscopy (SEM) evaluation

For each experimental group, the fixation of PONC complexes was performed in 2.5% glutaraldehyde/0.1 M phosphate-buffered saline (PBS) and prepared for conventional SEM evaluation. After washing of fixed samples in 0.1 M PBS, postfixation was done in 1% osmium tetroxide/0.1 M PBS. Finally, the samples were washed in 0.1 M PBS, and dehydrated in ascending ethanol series. The dried samples were put on aluminum stubs and, then, covered with gold. The SEM images were observed at low accelerating voltage (3-10 kV) using an SEM microscope (Philips XL-30-CP).

### RNA isolation and real-time polymerase chain reaction (real-time PCR) 

At the end of the culture period, the PONC complexes and PCOS oocytes (experimental and control groups, respectively) were collected. Total RNA was extracted using the RNeasy Mini Kit (Roche Molecular Biochemicals, Mannheim, Germany) and stored at -80°C. The cDNA was synthesized using the cDNA kit (Thermo Scientific, EU) in accordance with the manufacturer's instructions at 42°C for 60 min, and stored at -20°C.

Real-time PCR was used to quantify the mRNA transcript levels of the mitochondrial transcription factor A* (TFAM)* and *Cx43* genes. Primer pairs for amplifying the *TFAM* and *Cx43* genes were designed using GenBank from the National Center for Biotechnology Information. The primer sequences are shown in table I. Glyceraldehyde-3-phosphate dehydrogenase (*GAPDH)* was used as a housekeeping gene. Real time thermal cycler (Applied Biosystems, Foster City, USA) was used for analyzing gene expression. QuantiTect SYBR Green RT-PCR kit (Applied Biosystems, USA) was also employed for amplifying the targeted genes. Amplification of the reference and target genes was performed in the same run, for each sample. The thermal protocol of real-time PCR was programed as: the holding step at 95°C for 5 min, cycling step at 95°C for 15 sec, 58°C for 30 sec, and 72°C for 15 sec. The melt curve step was performed as 95°C (15 sec), 60°C (1 min), and 95°C (15 sec). The method of ΔΔCT was used to determine the relative quantity of the target genes. All experiments of real-time PCR were done five times.

**Table 1:** Designed primer sequences used for real-time PCR


**Genes**	**Primer pair sequence (5'-3')**	**Annealing temperature (°C)**	**Product length (bp)**	**Size (bp)**
	F 5'AGTGATCTCATCCGTCGAAG3'	58.9	21
* TFAM*	R 5'CTCCGTTCCAGTTCTTAAGCA3'	58.6	260	21
		F 5'CGCTGTAACACTCAACAACCC3'		59.8	21
* Cx43*	R 5' TTGCCGTGTTCTTCAATCCCA 3'	59.3	275	21
		F 5'CAAGGTCATCCATGACAACTTTG3'		58.2	23
* GAPDH* (endogenous)	R 5'GTCCACCACCCTGTTGCTGTAG3'	63.1	183	22
	*TFAM*: Mitochondrial transcription factor A, *Cx43*: Connexin-43, *GAPDH*: Glyceraldehyde-3-phosphate dehydrogenase, Bp: Base pair				

### Ethical considerations

All investigations conformed to the ethical and humane principles of research and were approved by Guilan University of Medical Science Committee on the Use and Care of Animals (Code: IR.GUMS.REC.1399.255).

### Statistical analysis

All experiments were repeated five times and data were expressed as mean ± S.D. $\chi^{2}$ , one-way ANOVA, and Tukey's post-hoc tests were used to analyze differences among the groups and gene expression. The statistical analysis was performed using the Statistical Package for the Social Sciences version 20 (SPSS, IBM, Armonk, NY, USA). P < 0.05 was considered statistically significant.

## 3. Results 

### PCOS ovaries evaluation

**Figure 2:**
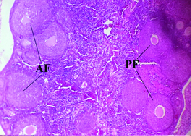
Ovarian histological section from a PCOS mouse ( × 10 magnification). The number of preantral and atretic follicles was higher in the PCOS ovary. AF: Atretic follicle, PF: Preantral follicle.

In the PCOS mice, the irregular estrous cycles were confirmed along with the restriction to estrous stages upon estradiol valerate treatment. The histological examinations showed that the number of pre-antral follicles was higher in the PCOS mice. In addition, atretic and cystic follicles were observed in these mice (n = 6 ovaries) and their ovaries contained fewer corpora lutea (Figure 2). The evaluation of steroid hormones showed that the serum testosterone and luteinizing hormone levels were higher in the estradiol valerate-treated mice (p = 0.04) at 60 days. The serum follicle-stimulating hormone level did not change after treatment with estradiol valerate.

In the experimental group, following co-culture of PCOS oocytes with normal GCCs, significant reaggregation of cells and oocytes was observed. These reconstructed complexes were formed as PONC complexes. The rate of reconstruction was 79.8%. The evaluation of the maturation rate of the PONC complex PCOS oocytes in the experimental group and of the PCOS oocytes from the control group showed that the nuclear maturation rate of PONC complexes was significantly higher compared to the control group (Table II, p = 0.01).

**Figure 3:**
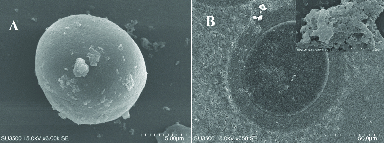
Scanning electron microscopy images of reconstructed PONC complexes after 12 hr (A) and 24 hr (B) of co-culture.

After co-culture, the PCOS oocytes were surrounded by multiple layers of strictly packaged GCCs, as shown in the SEM images (Figure 3).

The molecular analysis showed that the expression rates of the *TFAM* gene in the experimental and control groups were 0.460 ± 0.090 and 0.025 ± 0.005, respectively. The rate of *TFAM* gene expression was significantly higher in the experimental group (PONC complexes) in comparison to the control group (p = 0.03). In addition, *Cx43* gene expression was also higher in the experimental group (PONC complexes) (4.28 ± 0.57) compared with the control group (0.96 ± 0.12, p < 0.01) (Table III). The high expression of the *Cx43* gene could confirm the connection between PCOS oocytes and normal GCCs.

**Table 2:** Maturation rate of PCOS oocytes co-cultured with normal GCCs


	**Nuclear maturation of oocytes**			**p-value**
	** Oocyte (N)**	**GV + Deg**	**GVBD**	**MII**	
**Experimental group**		125	14.0 ± 1.2 (11.20)	18.0 ± 2.5 (14.40)	93.0 ± 7.7 (74.40)	
**Control group**	208	61.0 ± 6.2 (29.32)	26.0 ± 3.7 (12.50)	121.0 ± 8.9 (58.17)	0.008
Data are expressed as Mean ± SD and percentage (%). Chi-square test was used. GV: Germinal vesicle, Deg: Degenerative, GVBD: Germinal vesicle breakdown, MII: Metaphase II					

**Table 3:** The expression of the *TFAM* and *Cx43* genes in the experimental (PONC complexes) and control groups


**Groups**	*TFAM* gene	*Cx43* gene
**Experimental group**	0.460 ± 0.090	4.28 ± 0.57
**Control group**	0.025 ± 0.005	0.96 ± 0.12
**p-value**	0.03	0.0001
Data are expressed as Mean ± SD. Chi-square test was used. *TFAM*: Mitochondrial transcription factor A, *Cx43*: Connexin43		

## 4. Discussion

The maturation medium and IVM procedure strongly affected the oocyte quality and maturation. The present study showed that normal GCCs could interact with PCOS oocytes, with a gap junction created among them, and that as a result, PCOS oocyte maturation rates could improve. It seems that co-culturing with normal GCCs provides a suitable `niche' for PCOS oocyte maturation like the ovary. Therefore, after co-culture, the maturation rate of PCOS oocytes and the profile of gene expression improved. These results are reasonable because the dialogue between oocytes and the surrounding cells plays a major role in progressing oocyte meiosis and their developmental potential (14).

It is possible that some molecules from normal GCCs contribute to the growth potential of PCOS oocytes under in vitro conditions. Given that females with PCOS have a weak response to ovarian stimulation and have lower oocyte maturation competence (7), improving the medium efficiency for IVM protocols is important. The abnormal function of GCs in women with PCOS has been reported (16). In addition, the expression of the growth differentiation factor-9 gene is lower in GCCs from patients with PCOS (16). Growth differentiation factor-9 plays a critical role in promoting GC mitosis (17), maintaining and developing the gap junction (increased *Cx43* gene expression) between oocytes and adjacent GCs (18), generating luteinizing hormone, and synthesizing cyclic adenosine monophosphate (16, 19). Therefore, as the results of the present study show, co-culture with normal GCCs can improve in vitro maturation of PCOS oocytes. In accordance with previous studies, the results of this study show that *Cx43* gene expression was higher in the co-culture system between normal GCCs and PCOS oocytes. In addition, the expression of the *TFAM* gene, which is one of the genes representing the quality of the oocyte, was higher in the PCOS oocytes during co-culture. It has been observed that cumulus cells are difficult to detach (20); therefore, in this study they were only cultured for 24-48 hr before adding the PCOS oocytes. This prevented the attachment of the GCCs to the plate bottom and allowed interaction with and connection to the PCOS oocytes.

It has been reported that the cleavage rate of embryos resulting from matured oocytes in a co-cultured condition with cumulus cells can be higher in comparison to matured denuded oocytes (21). In one study, a novel co-culture system was described regarding preantral follicles along with antral follicles. This efficacious co-culture system promoted the development of small preantral follicles (11). This research was in line with the present study's results that the PCOS oocyte quality and meiotic progress was improved in the co-culture with normal GCCs. It has also been reported that the rate of mouse blastocyst formation can be improved in co-culture with cumulus cells (20). However, in a study by Lin and colleagues co-culture of oocytes with GCs did not have a positive effect on mice oocyte maturation (22).

It has been found that some substances are produced by GCs cultured in vitro. These substances could inhibit or delay the meiotic maturation of oocytes (23). The inhibitory effects of GCs on oocyte maturation were observed in a study on cows (24). Another study found that gap junctions among oocytes and cumulus cells could transfer cAMP among cells and the accumulation of cAMP in the oocytes resulted in meiosis inhibition (23). Therefore, there are still contradictory results in relation to the co-culture effects of GCs on oocyte maturation.

It seems plausible that soluble factors, such as the extra-cytoplasmic matrix or extracellular medium, can affect the formation of an oocyte-cumulus cell complex (25). The co-culture system, and consequently the connection between oocytes and GCs, may allow better coordination between nuclear and cytoplasmic maturation, which promotes maturation potential. Therefore, the proportion of matured oocytes is higher compared with ones in maturation media without GCs (26). It has also been demonstrated that an extensive production and reorganization of organelles and the replication of the mitochondrial genome occur during oocyte maturation. These alterations are vital for oocyte cytoplasmic maturation (25). Mitochondrial function is correlated with the mitochondrial DNA. During oocyte maturation, the amount of mitochondrial DNA becomes significantly larger. *TFAM* is known as an important factor in regulating mitochondrial DNA transcription and replication. Therefore, the relative expression of the *TFAM* gene and the developmental competence of oocytes are associated (25). In the present study, *TFAM* gene expression was significantly higher in PCOS oocytes co-cultured with normal GCCs. Therefore, it could be concluded that co-culture with normal GCCs may promote cytoplasmic maturation and developmental competence of PCOS oocytes.

## 5. Conclusion

The co-culture of PCOS oocytes with normal GCCs appears to improve PCOS-related abnormal follicular development. In addition, the connection among PCOS oocytes and GCCs, the higher levels of *Cx43* and *TFAM* gene expression, and the improved maturation of PCOS oocytes after co-culture suggest that the co-culture system using normal GCCs might be a better method for IVM protocols than adding different external factors. This may result in the promotion of assisted reproduction techniques.

##  Conflict of Interest

The authors have no conflict of interest to disclose.
